# Integrative Analyses of mRNA Expression Profile Reveal *SOCS2* and *CISH* Play Important Roles in *GHR* Mutation-Induced Excessive Abdominal Fat Deposition in the Sex-Linked Dwarf Chicken

**DOI:** 10.3389/fgene.2020.610605

**Published:** 2021-01-14

**Authors:** Genghua Chen, Jiahui Chen, Jingwen Wu, Xueyi Ren, Limin Li, Shiyi Lu, Tian Cheng, Liangtian Tan, Manqing Liu, Qingbin Luo, Shaodong Liang, Qinghua Nie, Xiquan Zhang, Wen Luo

**Affiliations:** ^1^Department of Animal Genetics, Breeding and Reproduction, College of Animal Science, South China Agricultural University, Guangzhou, China; ^2^Key Lab of Chicken Genetics, Breeding and Reproduction, Ministry of Agriculture, South China Agricultural University, Guangzhou, China

**Keywords:** sex-linked dwarf chicken, abdominal fat deposition, *SOCS2*, *CISH*, differentially expressed gene

## Abstract

Sex-linked dwarf (SLD) chicken, which is caused by a recessive mutation of the growth hormone receptor (*GHR*), has been widely used in the Chinese broiler industry. However, it has been found that the SLD chicken has more abdominal fat deposition than normal chicken. Excessive fat deposition not only reduced the carcass quality of the broilers but also reduced the immunity of broilers to diseases. To find out the key genes and the precise regulatory pathways that were involved in the *GHR* mutation-induced excessive fat deposition, we used high-fat diet (HFD) and normal diet to feed the SLD chicken and normal chicken and analyzed the differentially expressed genes (DEGs) among the four groups. Results showed that the SLD chicken had more abdominal fat deposition and larger adipocytes size than normal chicken and HFD can promote abdominal fat deposition and induce adipocyte hypertrophy. RNA sequencing results of the livers and abdominal fats from the above chickens revealed that many DEGs between the SLD and normal chickens were enriched in fat metabolic pathways, such as peroxisome proliferator-activated receptor (PPAR) signaling, extracellular matrix (ECM)-receptor pathway, and fatty acid metabolism. Importantly, by constructing and analyzing the *GHR*-downstream regulatory network, we found that suppressor of cytokine signaling 2 (*SOCS2*) and cytokine-inducible SH2-containing protein (*CISH*) may involve in the *GHR* mutation-induced abdominal fat deposition in chicken. The ectopic expression of *SOCS2* and *CISH* in liver-related cell line leghorn strain M chicken hepatoma (LMH) cell and immortalized chicken preadipocytes (ICP) revealed that these two genes can regulate fatty acid metabolism, adipocyte differentiation, and lipid droplet accumulation. Notably, overexpression of *SOCS2* and *CISH* can rescue the hyperactive lipid metabolism and excessive lipid droplet accumulation of primary liver cell and preadipocytes that were isolated from the SLD chicken. This study found some genes and pathways involved in abdominal fat deposition of the SLD chicken and reveals that *SOCS2* and *CISH* are two key genes involved in the *GHR* mutation-induced excessive fat deposition of the SLD chicken.

## Introduction

Broiler consumption accounts for a large part of global meat consumption. Sex-linked dwarf (SLD) chicken is caused by deletion or point mutations of the growth hormone receptor (*GHR*) gene located in the Z chromosome (Tahara et al., 2009). Compared with normal chicken, the SLD chicken has a variety of phenotypic and physiological alteration and has been widely used in broiler breeding due to lower basal metabolism, heat stress resistance, and higher feed conversion rate. Besides, homozygous SLD chicken has smaller muscle fiber diameter and higher intramuscular fat deposition, which plays an essential role in meat quality (Knížetová, 1993; Melesse et al., 2013; Ye et al., 2014; Ferdaus et al., 2016; Luo et al., 2016; Cui et al., 2019). Furthermore, the SLD chicken can be used as an important model organism to study human inherited diseases caused by *GHR* gene mutations. The symptoms of those diseases are similar to the SLD chicken, which has short shape, obesity, high level of serum growth hormone (GH), and low level of serum IGF-1 (Berg et al., 1993).

Excessive abdominal fat deposition is becoming more common in the broiler industry with the increase of growth rate and the popularization of large-scale production. Excessive fat has often been discarded because of processing difficulty and unhealthy diet. Additionally, excessive fat can also reduce feed conversion rate, carcass yield, and reproductive performance (Lagarrigue et al., 2006; Zhang et al., 2017, 2018). Therefore, reducing excessive fat deposition has become an important goal of broiler breeding. It is worth noting that mutation or abnormal expression of *GHR* often results in lipid metabolism disorder. The ablation of *GHR* mRNA, deletion, and mutations in the *GHR* exons are related to human obesity, such as the increase of abdominal fat and subcutaneous fat content (Erman et al., 2011; Glad et al., 2015). Moreover, patients with Laron syndrome caused by the *GHR* mutations have significant trunk obesity and body composition change (Ginsberg et al., 2009). Similar to Laron syndrome phenotype, global *GHR* knockout mice (*GHR*^–/–^) showed GH tolerant, obese, highly sensitive to insulin, enhanced glucose assimilation, and extended longevity (Masternak et al., 2012). The lipolytic/antilipogenic effect of GH is disrupted in *GHR*^–/–^ mice, which is effective in suppressing fat consumption. The whole adipose tissue mass of *GHR*^–/–^ mice was significantly increased compared with littermate negative control (Berryman et al., 2009). Different from the global *GHR*-KO mice, fat tissue specific *GHR*-knockout (FaGHR-KO) mice were larger than the control group. The white fat, brown fat, and adipocyte sizes were all significantly increased in the FaGHR-KO mice (List et al., 2019). Adipocyte specific-knockout mice showed increased sensitivity to dietary obesity and are able to protect the liver from high-fat diet (HFD)-induced liver injury by trapping free fatty acids (Ran et al., 2019). In addition, skeletal muscle specific *GHR*-KO mice increased insulin sensitivity in response to HFD intake, suggesting a link between the GHR signaling pathway in the skeletal muscle, liver, and adipose tissue (Vijayakumar et al., 2011).

The reaction of several downstream signaling pathways of the GH–GHR axis has been studied in case of *GHR* mutations or knockout. A trimolecular complex was formed when GH was bound to GHR, leading to GHR conformation transitions and activated downstream GHR-mediated signaling pathways. *GHR* mutations result in low effective or even abolished interaction of GH and GHR and further suppress secretion of IGF-1 by the GH–GHR–IGF-1 axis (Filopanti et al., 2011; Lin et al., 2018). GH binding to GHR would activate JAK2 and the members of the signal transducer and activator of transcription (STAT), STAT1, STAT3, and STAT5 (Dehkhoda et al., 2018). The mitogen-activated protein kinase (MAPK) pathway and the phosphoinositide-3 kinase (PI3K) and protein kinase B (AKT) pathway are also the downstream pathways of GH–GHR signaling (Rosenfeld et al., 2007; Lin et al., 2018). Targeted disruption of *GHR* eliminated STAT5a and STATb signal transduction (Rosenfeld et al., 2007). STAT5 cannot be activated in *GHR*-KO mice, causing the enhancement of lipogenesis and obesity (Chhabra et al., 2019). In the liver of fasted *GHR*-KO pigs, phosphorylation of STAT5 was significantly decreased, whereas the phosphorylation of glycogen synthase 3 beta, PI3K, and AKT was increased (Hinrichs et al., 2018). It was shown that AKT is an essential regulator of peroxisome proliferator-activated receptor (PPAR) γ (Peng, 2003). AKT can regulate adipogenesis by interacting with the mTOR pathway, and it can also inhibit FOXO members to increase PPARγ expression and promote adipocyte differentiation (Cai et al., 2016; Pan et al., 2017). mTOR is active in two compounds, mTORC1 and mTORC2. In several tissues of *GHR*-KO mice, mTORC1 was inhibited, whereas mTORC2 was elevated to regulate AKT and enhance lipid synthesis (Fang et al., 2018). Though it has been known that the chicken with *GHR* mutation or deletion has more abdominal fat deposition than normal chicken, the internal mechanism of *GHR* mutation-induced excessive fat deposition remains unknown. Therefore, the main purpose of this study was to find out the key genes and the precise regulatory pathways that were involved in the *GHR* mutation-induced excessive fat deposition in the SLD chickens.

## Materials and Methods

### Animal Experimental Procedures

A total of 12 female dwarf type Xinghua chickens (the SLD chicken, with *GHR* gene exon 5 a T/C mutation) were randomly distributed to the HFD group, which was fed a diet consisting of 40% carbohydrate, 25% fat, and 20% protein, and the normal diet group, which was fed a diet consisting of 41% carbohydrate, 5% fat, and 22% protein (*n* = 6 in each group). Those 8-week-old SLD chickens with similar body weight were fed for 2 weeks and weighed before slaughter. In addition, another 12 purebred female Xinghua chickens (normal chicken) were treated in the same way as the SLD chicken. Determination on abdominal fat weight and abdominal fat rate was carried out as described previously (Chen et al., 2019). The tissues of liver and abdominal fat were immediately frozen in liquid nitrogen and stored at −80°C until use. All animal work was approved and performed in accordance with the regulations and guidelines of the Institutional Animal Care and Use Committee of the South China Agricultural University (approval number: SCAU#0017; 21 November 2017).

### H&E Staining and Adipocyte Size Calculation

Abdominal fat was fixed with 4% paraformaldehyde and embedded in paraffin, and 12-μm-thick serial sections were made. Then, sections were performed H&E staining according to standard protocols. Microscopic observation and photograph were taken with Leica DM2500 microscope (Leica, Wetzlar, Germany). At least five visual fields were randomly selected for each section, and at least 20 cells were selected for each visual field. The area and diameter of adipocyte were calculated using Nikon Eclipse Ti microscope and NIS-Elements BR software (Nikon, Tokyo, Japan).

### Plasmid Construction and Small Interfering RNA

Suppressor of cytokine signaling 2 (*SOCS2*) and cytokine-inducible SH2-containing protein (*CISH*) coding sequences were amplified from chicken liver cDNA by PCR using specific primers (Supplementary File 1). The full-length coding regions of chicken *SOCS2* and *CISH* were constructed into pcDNA3.1 vector (Invitrogen). Specific small interfering RNAs of *SOCS2* and *CISH* were obtained from RiboBio, and non-specific siRNA was used as the control.

### Cell Culture and Transfection

Liver-related cell line leghorn strain M chicken hepatoma (LMH) cell and immortalized chicken preadipocytes (ICP) were cultured in high-glucose Dulbecco’s modified Eagle’s medium (Gibco, Carlsbad, CA, United States) with 10% fetal bovine serum and 0.2% penicillin/streptomycin at the condition of 37°C with 5% CO_2_. ICP cells were induced to differentiation by adding 160 μM sodium oleate (Sigma Life Science, St. Louis, MO, United States). Plasmid DNA and siRNA transfection were carried out using the transfection reagent Lipofectamine 3000 (Invitrogen Corporation, Carlsbad, CA, United States) following the manufacturer’s protocol.

### Quantitative Real-Time PCR

RNA from tissues or cells was isolated using RNAiso reagent (Takara, Otsu, Japan). Reverse-transcription reactions were using PrimeScript RT reagent Kit with gDNA Eraser (Takara). The relative mRNA expression levels of genes were determined by using real-time quantitative PCR (qPCR) with SYBR Green. The specific primers are shown in Supplementary File 1. The 2^–ΔΔCt^ method was used to calculate gene expression with β-actin (for liver tissues and LMH cells) or GAPDH (for abdominal fats and ICP cells).

### Gene Expression Profiling

Three livers and abdominal fats from each of the four treatment groups were selected for RNA sequencing. RNA quantity and quality were evaluated on an Agilent 2100 Bioanalyzer (Agilent Technologies, Waldbronn, Germany), and RNA integrity was further examined using agarose gel electrophoresis. High-throughput RNA-seq was performed on the BGISEQ-500 platform (BGI, Wuhan, China). Significance was accepted at adjusted | log2FC| ≥ 0.5, *P* ≤ 0.001. All the sequence data have been deposited in NCBI’s Gene Expression Omnibus (GEO^1^) and are accessible through GEO series accession numbers GSE129840 and GSE128340.

### Oil Red O Staining and Quantification

After 48 h of transfection, Oil Red O staining was carried out in ICP cells. Briefly, cells were washed with PBS for 5 min and then fixed with 4% paraformaldehyde for 10 min. The cells were stained with Oil Red O working solution (Solarbio, Beijing, China). The cells were observed and photographed using a microscope (Leica). The Oil Red O dyes were isolated using isopropanol solution containing 4% Non-idet P-40. Concentration was quantified by Model 680 Microplate Reader (Bio-rad, CA, United States) at 510 nm.

### Bioinformatics Analysis

The Venn diagram was calculated and drawn by a web-based software^2^. Gene ontology (GO) analysis of the enriched genes was performed using the web-based Metascape (a gene annotation and analysis resource^3^ (Zhou et al., 2019). Kyoto Encyclopedia of Genes and Genomes (KEGG) pathway analysis of differentially expressed genes (DEGs) was carried out for functional analyses^4^ (Kanehisa, 2000; Kanehisa et al., 2017).

### Statistical Analysis

All data are reported as mean ± SEM. The difference between two groups was evaluated using independent sample *t*-test. The differences among multiple groups were evaluated using Duncan’s multiple range test. *P* < 0.05 was considered to be statistically significant. All experiments were carried out at least three times. All statistical analyses were performed using SPSS 18.0 for Windows (SPSS, Inc., Chicago, IL, United States).

## Results

### The SLD Chickens Have More Abdominal Fat Deposition and Larger Adipocytes Size Than Normal Chickens

**FIGURE 1:**
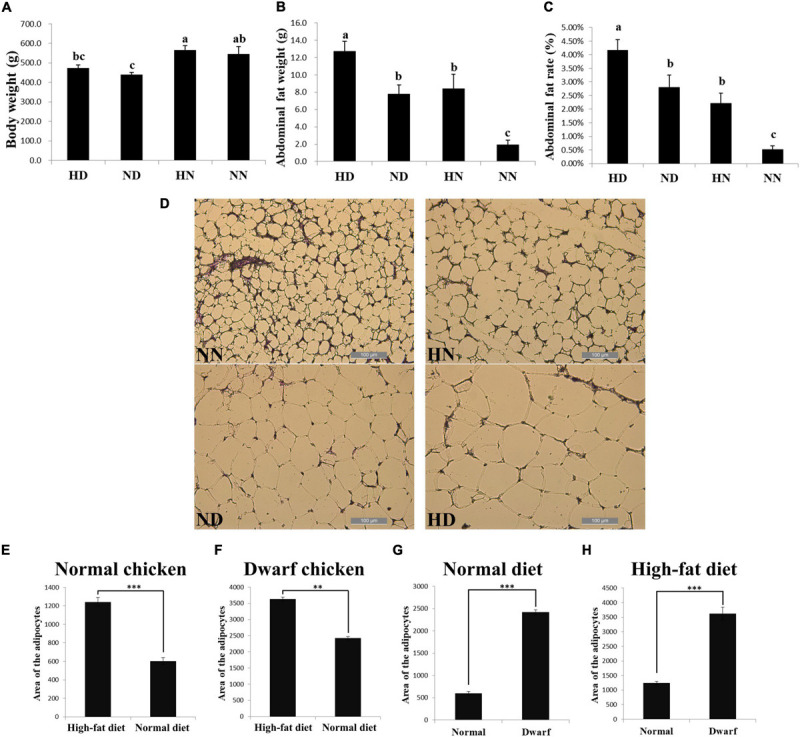
The sex-linked dwarf (SLD) chickens have more abdominal fat deposition and larger adipocytes size than normal chickens. **(A)** Body weight of the SLD chicken fed with high-fat diet (HD) and normal diet (ND) and normal chicken fed with high-fat diet (HN) and normal diet (NN). **(B)** Abdominal fat weight of the HD, ND, HN, and NN chickens. **(C)** Abdominal fat rate of the HD, ND, HN, and NN chickens. **(D)** Micrograph of abdominal fat cross-section from the NN, HN, ND, and HD chickens. Bar: 200 μm. **(E)** Adipocytes area of normal chicken fed with high-fat diet and normal diet. **(F)** Adipocytes area of the SLD chicken fed with high-fat diet and normal diet. **(G)** Adipocytes area from normal chicken and the SLD chicken fed with normal diet. **(H)** Adipocytes area from normal chicken and the SLD chicken fed with high-fat diet. The data are mean ± SEM with at least three samples (*n* ≥ 3/treatment group). The different lowercase letters above columns indicate significant differences among HD, HN, ND, and NN chickens (*P* < 0.05) by Duncan’s multiple range test. Independent sample *t*-test was used to analyze the statistical differences between two groups. ^∗∗^*P* < 0.01; ^∗∗∗^*P* < 0.001.

To investigate the difference of abdominal fat deposition between the SLD and normal chickens and test the effect of HFD on these two kinds of chicken breeds, we fed the SLD chickens and normal chickens with HFD and normal diet. After 2 weeks of feeding, HFD fed SLD chicken (HD), HFD fed normal chicken (HN), normal diet fed SLD chicken (ND), and normal diet fed normal chicken (NN) were weighed and conducted carcass determination. Results showed that the body weight of the SLD chickens was significantly lower than that of normal chickens under the same diet, whereas the body weight of either chicken breeds was not significantly affected by HFD (Figure 1A). However, HFD can significantly increase the abdominal fat weight and abdominal fat rate of chickens, and the abdominal fat weight and abdominal fat rate of the SLD chicken were significantly higher than those of normal chicken under the same diet (Figures 1B,C). Next, we analyzed the adipocytes size between chickens. The HFD can significantly increase the adipocyte size of the SLD chicken and normal chicken (Figures 1D–F). Compared with normal chicken, larger adipocytes area was observed in the SLD chicken in both HFD and normal diet (Figures 1G,H). These results demonstrated that the SLD chickens have more abdominal fat deposition and larger adipocytes size than normal chickens, and that HFD can increase abdominal fat deposition and enlarge adipocytes size in chicken.

### DEGs Analyses in the Liver Between the SLD Chicken and Normal Chicken

**FIGURE 2:**
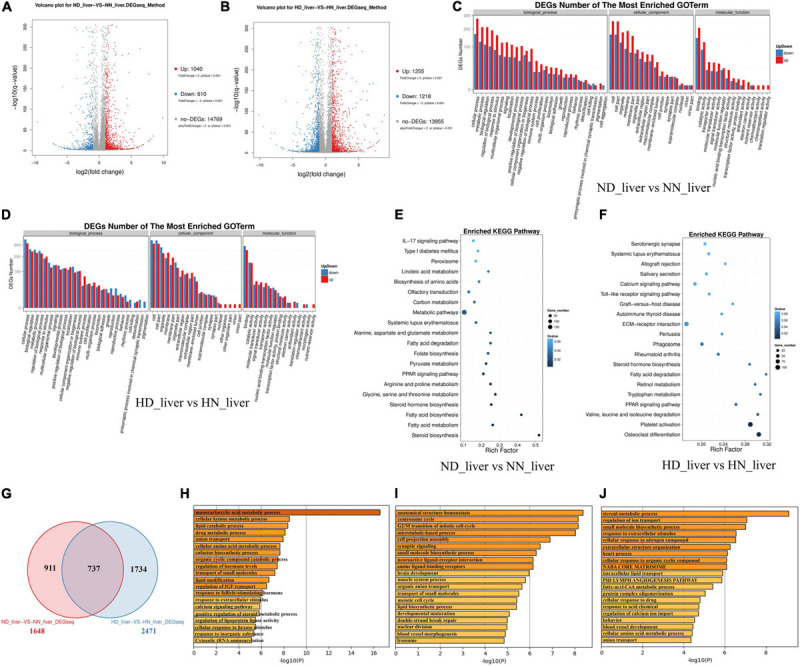
Differentially expressed genes (DEGs) analysis in the liver between the sex-linked dwarf (SLD) chicken and normal chicken. **(A)** Scatter plot of DEGs between normal diet fed SLD chicken (ND) and normal diet fed normal chicken (NN) in the liver. **(B)** Scatter plot of DEGs between high-fat diet fed SLD chicken (HD) and high-fat diet fed normal chicken (HN) in the liver. **(C)** Gene Ontology enrichment of DEGs between ND and NN chickens in the liver. **(D)** Gene Ontology enrichment of DEGs between HD and HN chickens in the liver. **(E)** Enriched Kyoto Encyclopedia of Genes and Genomes pathway of DEGs between ND and NN chickens in the liver. **(F)** Enriched Kyoto Encyclopedia of Genes and Genomes pathway of DEGs between HD and HN chickens in the liver. **(G)** Venn diagram analysis of DEGs in the liver between the SLD chicken and normal chicken under different diet conditions. **(H)** Metascape functional enrichment analysis of common DEGs between ND-vs-NN and HD-vs-HN chickens in the liver. **(I)** Metascape functional enrichment analysis of DEGs specific differentially expressed in ND_liver-VS-NN_liver (911 genes). **(J)** Metascape functional enrichment analysis of DEGs specific differentially expressed in HD_liver-VS-HN_liver (1,734 genes).

Considering that the liver is the main organ of lipid metabolism and plays an essential role in the digestion, absorption, synthesis, decomposition, and transportation of lipids, we collected the livers of HD, HN, ND, and NN chickens for RNA sequencing to find DEGs and signal pathways involved in *GHR* mutation-induced difference of lipid metabolism. A total of 1,040 genes were upregulated, and 610 genes were downregulated in ND chicken compared with NN chicken (Figure 2A and Supplementary File 2), whereas a total of 1,255 genes were upregulated, and 1,218 genes were downregulated in HD chicken compared with HN chicken (Figure 2B and Supplementary File 3). GO Enrichment Analysis showed that the DEGs between the SLD chicken and normal chicken were mainly related to the metabolic process and developmental process (Figures 2C,D). KEGG pathway analysis showed that the enriched pathways of DEGs between ND and NN chickens included many lipid metabolism and adipose deposition pathways, such as metabolic pathways, linoleic acid metabolism, fatty acid degradation, PPAR signaling pathway, steroid hormone biosynthesis, fatty acid biosynthesis, fatty acid metabolism, and steroid biosynthesis (Figure 2E). On the other hand, the DEGs between HD and HN chickens were also enriched in the pathways involved in lipid metabolism and adipocyte development, such as PPAR signaling pathway, fatty acid degradation, steroid hormone biosynthesis, calcium signaling pathway, and extracellular matrix (ECM)-receptor interaction (Figure 2F). Furthermore, we identified 737 genes differentially expressed between the two kinds of chicken breeds not only under the HFD but also under the normal diet (Figure 2G and Supplementary File 4). The functions of these 737 DEGs were largely related to lipid metabolism, such as lipid catabolic process and lipid modification (Figure 2H). A total of 911 genes were identified specific differentially expressed between the SLD chicken and normal chicken under normal diet, and the functions of these DEGs were mainly related to cell cycle and lipid biosynthetic process (Figure 2I). A total of 1,734 DEGs were specific differentially expressed between the SLD chicken and normal chicken under HFD, and their functions were mainly enriched in metabolic process and lipid transport (Figure 2J). Therefore, these results not only found the DEGs of the liver between the SLD chicken and normal chicken but also obtained the potential signaling pathways and cellular processes involved in the *GHR* mutation-induced liver lipid metabolism difference between the SLD chicken and normal chicken.

### DEGs Analyses in the Abdominal Fat Between the SLD Chicken and Normal Chicken

**FIGURE 3:**
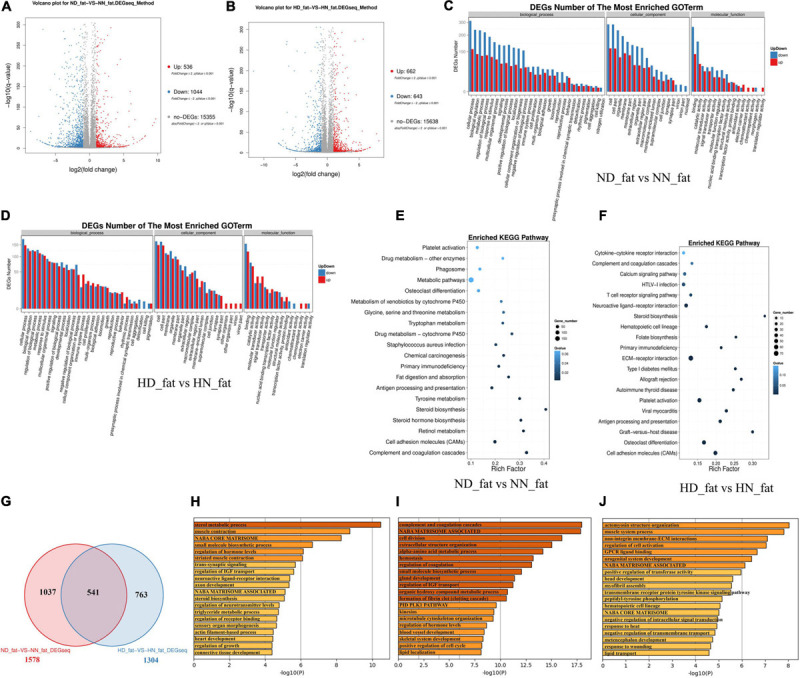
Differentially expressed genes analyses in the abdominal fat between the sex-linked dwarf (SLD) chicken and normal chicken. **(A)** Scatter plot of differentially expressed genes (DEGs) between normal diet fed SLD chicken (ND) and normal diet fed normal chicken (NN) in the abdominal fat. **(B)** Scatter plot of DEGs between high-fat diet fed SLD chicken (HD) and high-fat diet fed normal chicken (HN) in the abdominal fat. **(C)** Gene Ontology enrichment of DEGs between ND and NN chickens in the abdominal fat. **(D)** Gene Ontology enrichment of DEGs between HD and HN chickens in the abdominal fat. **(E)** Enriched Kyoto Encyclopedia of Genes and Genomes pathway of DEGs between ND and NN chickens in the abdominal fat. **(F)** Enriched Kyoto Encyclopedia of Genes and Genomes pathway of DEGs between HD and HN chickens in the abdominal fat. **(G)** Venn diagram analysis of DEGs in the abdominal fat between the SLD chicken and normal chicken under different diet conditions. **(H)** Metascape functional enrichment analysis of common DEGs between ND-vs-NN and HD-vs-HN chickens in the abdominal fat. **(I)** Metascape functional enrichment analysis of DEGs specific differentially expressed in ND_fat-VS-NN_fat (1,037 genes). **(J)** Metascape functional enrichment analysis of DEGs specific differentially expressed in HD_fat-VS-HN_fat (763 genes).

In order to find DEGs involved in abdominal fat deposition and adipogenesis, we collected abdominal fat from HD, HN, ND, and NN chickens for RNA sequencing. A total of 536 genes were upregulated, and 1,044 genes were downregulated in fat between ND chicken and NN chicken (Figure 3A and Supplementary File 5). Under an HFD, a total of 622 genes were upregulated expression, and 643 genes were downregulated expression (Figure 3B and Supplementary File 6). GO analysis of DEGs between the SLD chicken and normal chicken under a normal diet found that these DEGs were related to metabolism process, developmental process, and growth (Figure 3C). DEGs under an HFD also exhibited similar biological process enrichment (Figure 3D). KEGG analysis of DEGs between ND and NN chickens showed that the mainly enriched pathways included metabolic pathway, cell adhesion molecules (CAMs), and fat digestion and absorption (Figure 3E), whereas calcium signaling pathway, ECM-receptor interaction, type I diabetes mellitus, and CAMs were included in the enriched KEGG pathways of DEGs between HD and HN chickens (Figure 3F). Furthermore, we identified 541 genes differentially expressed between the two kinds of chicken breeds not only under the HFD but also under the normal diet (Figure 3G and Supplementary File 7). The functions of these 541 DEGs were largely related to hormone metabolism and growth, such as sterol metabolic process, regulation of IGF transport, steroid biosynthesis, triglyceride metabolic process, and regulation of growth (Figure 3H). A total of 1,037 genes were identified specific differentially expressed between the two kinds of chicken breeds under the normal diet, and the functions of these DEGs were mainly involved in the cell proliferation and growth processes (Figure 3I). A total of 763 DEGs were specific differentially expressed between the two kinds of chicken breeds under HFD, and the functions of these DEGs were involved in signal transduction, development, and lipid transport (Figure 3J). Together, these results not only found the DEGs of the abdominal fat between the SLD chicken and normal chicken but also obtained the potential signaling pathways and cellular processes involved in the GHR mutation-induced fat deposition and adipogenesis.

### Integrative Analyses Reveal DEGs Involved in HFD-Induced Fat Deposition

**FIGURE 4:**
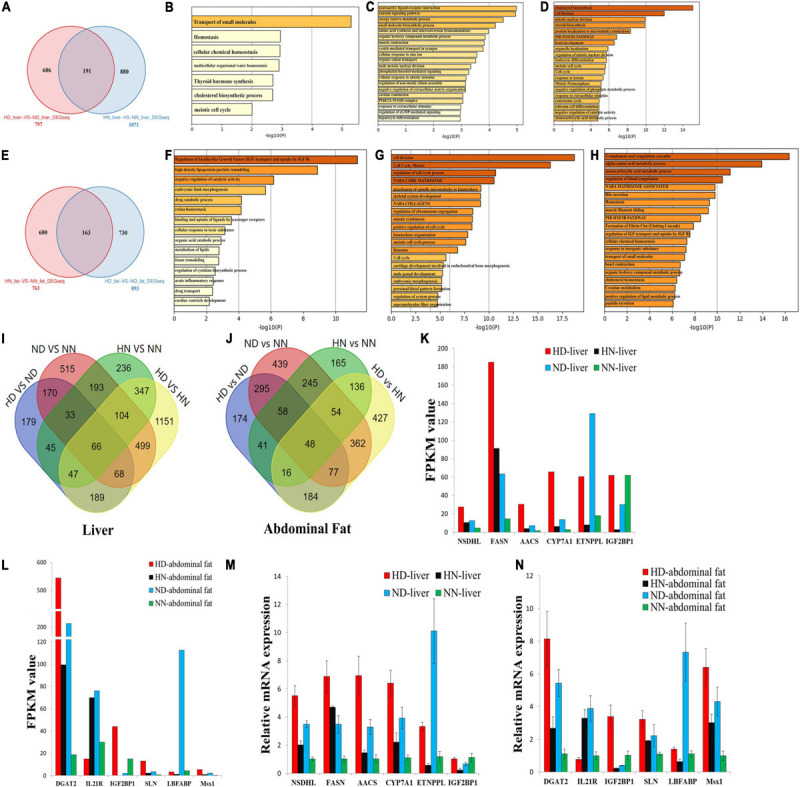
Integrative analyses reveal differentially expressed genes involved in high-fat diet (HFD)-induced fat deposition. **(A)** Specific and common differentially expressed genes (DEGs) between the sex-linked dwarf (SLD) chicken and normal chicken under the HFD and the normal diet (HD-vs-ND compared with HN-vs-NN) in the liver. **(B)** Metascape functional enrichment analysis of the common DEGs between HD-vs-ND and HN-vs-NN. **(C)** Metascape functional enrichment analysis of DEGs specific differentially expressed in the HD-vs-ND group. **(D)** Metascape functional enrichment analysis of DEGs specific differentially expressed in HN-vs-NN group. **(E)** Specific and common DEGs between the SLD chicken and normal chicken (HN-vs-NN compared with HD-vs-ND) in the abdominal fat. **(F)** Metascape functional enrichment analysis of the common DEGs between HN-vs-NN and HD-vs-ND. **(G)** Metascape functional enrichment analysis of DEGs specific differentially expressed in HN-vs-NN. **(H)** Metascape functional enrichment analysis of DEGs specific differentially expressed in HD-vs-ND. **(I)** Venn diagram analysis of DEGs among HD-vs-ND, ND-vs-NN, HN-vs-NN, and HD-vs-HN in the liver. **(J)** Venn diagram analysis of DEGs among HD-vs-ND, ND-vs-NN, HN-vs-NN, and HD-vs-HN in the abdominal fat. **(K)** The expression of six genes selected from the 66 common DEGs was determined by RNA-sequence. **(L)** The expression of six genes selected from the 48 common DEGs was determined by RNA-sequence. **(M)** The expression of six genes selected from the 66 common DEGs was determined by qPCR. **(N)** The expression of six genes selected from the 48 common DEGs was determined by qPCR. The data are mean ± SEM with three samples (*n* = 3/treatment group).

To further analyze the different effects of HFD on the SLD chicken and normal chicken, we screened out the common and specific DEGs between the SLD chicken and normal chicken. In the liver, we found a total of 191 common DEGs (Figure 4A and Supplementary File 8), which mean that HFD induces the differential expression of these genes not only in the SLD chickens but also in normal chickens. The functions of these common DEGs were involved in transport of small molecules, hemostasis, and cell cycle (Figure 4B). We also found that 606 DEGs were specific differentially expressed between HD and ND chickens (Figure 4A), and that the functions of these DEGs were related to metabolic processes and hepatocyte differentiation (Figure 4C). Besides, a total of 880 DEGs were specific differentially expressed between HN and NN chickens, and these DEGs were mainly enriched in cell cycle process (Figure 4D).

In abdominal fat, a total of 163 genes were not only differentially expressed between HN and NN chickens but also differentially expressed between HD and ND chickens (Figure 4E and Supplementary File 9). The functions of these common DEGs were related to IGF transport and lipid metabolism (Figure 4F). Additionally, the functions of the 600 DEGs specific differentially expressed between HN and NN fats were mainly enriched in cell cycle, such as cell division, mitotic, regulation of cell cycle process, regulation of chromosome segregation, positive regulation of cell cycle, and meiotic cell cycle process (Figure 4G). The functions of the 730 DEGs, which were specific differentially expressed between HD and ND fats, were related to multiple metabolic processes and homeostasis (Figure 4H).

Next, we compared the common and specific DEGs between different treatment groups in the liver and in the abdominal fat, respectively (Figures 4I,J). We found that a total of 66 DEGs were common differentially expressed in the liver among the four treatment groups (Figure 4I and Supplementary File 10), and that 48 DEGs were common differentially expressed in the abdominal fat among the four treatment groups (Figure 4J and Supplementary File 10). These common DEGs may play important roles in chicken abdominal fat deposition. To test the quality and accuracy of RNA-seq results, we selected several common DEGs, which are related to lipid metabolism and adipogenesis, in the liver and in the abdominal fat, respectively (Figures 4K,L). Then, we used qPCR to validate the expression of DEGs among the four treatment groups. Results showed that the qPCR data were consistent with RNA-seq data (Figures 4M,N), demonstrating that the RNA-seq results were highly reliable.

### *GHR*-Mediated Signaling Pathway Analyses Reveal Key Pathways and Genes Relative to *GHR* Mutation-Induced Fat Deposition

**FIGURE 5:**
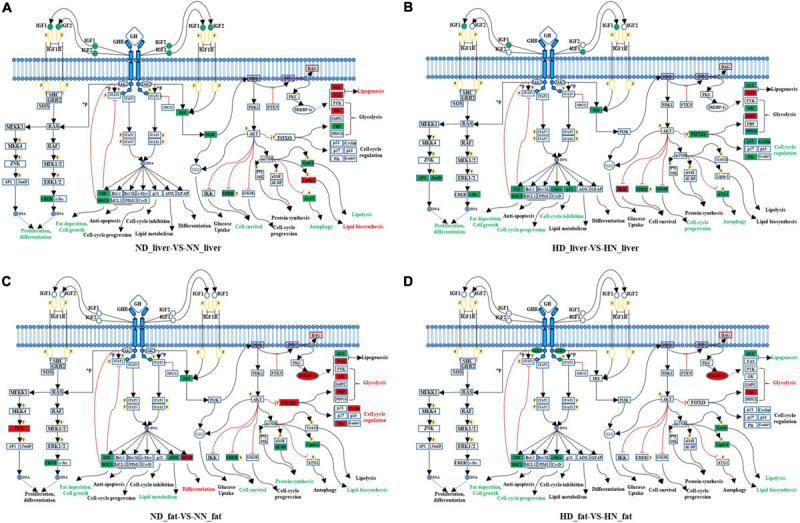
*GHR*-mediated signaling pathways analyses reveal key pathways and genes relative to *GHR* mutation-induced fat deposition. **(A)** Differentially expressed genes (DEGs) of the liver between normal diet fed sex-linked dwarf (SLD) chicken (ND) and normal diet fed normal chicken (NN) enriched in *GHR*-mediated signaling pathways. **(B)** DEGs of the liver between high-fat diet fed SLD chicken (HD) and high-fat diet fed normal chicken (HN) enriched in *GHR*-mediated signaling pathways. **(C)** DEGs of the abdominal fat between normal diet fed SLD chicken (ND) and normal diet fed normal chicken (NN) enriched in *GHR*-mediated signaling pathways. **(D)** DEGs of the abdominal fat between high-fat diet fed SLD chicken (HD) and high-fat diet fed normal chicken (HN) enriched in *GHR*-mediated signaling pathways. The red fonts or boxes indicate that the genes or biology functions were inhibited. The green fonts or boxes indicate that the genes or biology functions were promoted.

Growth hormone binds to GHR and activates a variety of downstream signaling pathway. In order to better understand how *GHR* mutation affects downstream signaling transduction, we constructed a *GHR*-mediated downstream genes interaction network, which includes JAK–STAT, PI3K–AKT–mTOR, FOXO signaling, GH–GHR–IGFs, and MAPK signaling pathways (Figure 5). Next, we used this network to map the DEGs between the SLD chicken and normal chicken. The DEGs between ND liver and NN liver were enriched in PI3K–Akt–mTOR signaling pathway, which regulated lipid biosynthesis, lipolysis, and cell autophagy (Figure 5A). Additionally, the DEGs between HD liver and HN liver were enriched in JAK–STAT, AKT, and FOXO signaling pathways. Notably, the function of these DEGs was mainly related to cell cycle regulation (Figure 5B). On the other hand, the DEGs between ND and NN abdominal fats were enriched in FOXO signaling pathway, and the functions of these DEGs were related to glycolysis, cell cycle, lipid biosynthesis, and lipid metabolism (Figure 5C). The DEGs between HD and HN abdominal fats were enriched in JAK–STAT signaling pathway, and the functions of these DEGs were related to lipogenesis, glycolysis, and cell cycle (Figure 5D). Notably, *SOCS2* and *CISH* were the only two genes that were differentially expressed in all of the four treatment groups, indicating their important roles on lipid metabolism and *GHR* mutation-induced fat deposition.

### *SOCS2* Inhibits Lipid Metabolism and Decreases Lipid Droplet Accumulation

**FIGURE 6:**
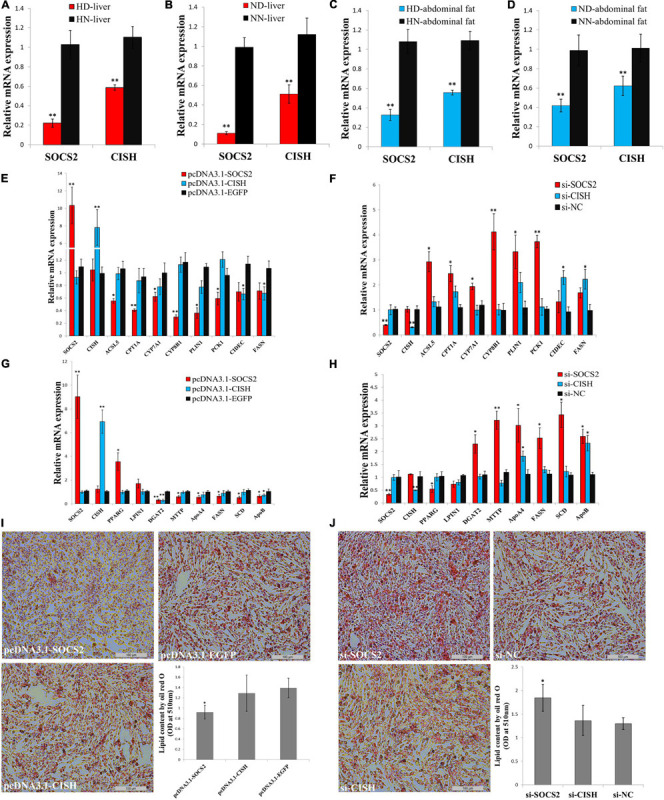
*SOCS2* inhibits lipid metabolism and decreases lipid droplet accumulation. **(A)** The mRNA expression of *SOCS2* and *CISH* in the liver of high-fat diet fed sex-linked dwarf chicken (HD) compared with high-fat diet fed normal chicken (HN). **(B)** The mRNA expression of *SOCS2* and *CISH* in the liver of normal diet fed sex-linked dwarf chicken (ND) compared with normal diet fed normal chicken (NN). **(C)** The mRNA expression of *SOCS2* and *CISH* in the abdominal fat of HD chicken compared with HN chicken. **(D)** The mRNA expression of *SOCS2* and *CISH* in the abdominal fat of ND chicken compared with NN chicken. **(E)** The expression of lipid metabolism-related differentially expressed genes (DEGs) after transfection of *SOCS2* or *CISH* in LMH cell. **(F)** The expression of lipid metabolism-related DEGs after transfection of si-SOCS2 or si-CISH in LMH cell. **(G)** The expression of fat deposition-related DEGs after transfection of *SOCS2* or *CISH* in ICP cell. **(H)** The expression of fat deposition-related DEGs after transfection of si-SOCS2 or si-CISH in ICP cell. **(I)** Representative images of Oil Red O staining (red) after overexpression of *SOCS2* or *CISH* in ICP cells; scale bar: 100 μm. **(J)** Representative images of Oil Red O staining (red) after inhibition of *SOCS2* or *CISH* in ICP cells; scale bar: 100 μm. The data are mean ± SEM with four samples (*n* = 4/treatment group). Independent sample *t*-test was used to analyze the statistical differences between groups. ^∗^*P* < 0.05; ^∗∗^*P* < 0.01.

In order to understand the roles of *SOCS2* and *CISH* on *GHR* mutation-induced abdominal fat deposition, we tested their functions in lipid metabolism and adipocyte differentiation *in vitro*. The expression of *SOCS2* and *CISH* was significantly downregulated in the liver and abdominal fat of dwarf chicken compared with normal chicken, no matter in an HFD or normal diet condition (Figures 6A–D). Next, we used LMH cell and ICP cell to test the function of *SOCS2* and *CISH in vitro*. To assess the function of *SOCS2* and *CISH* in lipid metabolism and fat deposition and find the potential downstream genes that can be affected by *SOCS2* and *CISH*, we selected several genes that were not only involved in lipid metabolism and fat deposition but also differentially expressed between the SLD chicken and normal chicken as candidate genes to define the status of lipid metabolism and fat deposition. In LMH cell, *SOCS2* overexpression significantly inhibited the expression of most of the lipid metabolism-related DEGs, whereas *CISH* overexpression can only inhibit the expression of *CIDEC* and *FASN* (Figure 6E). On the other hand, *SOCS2* knockdown significantly promoted the expression of most of the lipid metabolism-related DEGs, whereas *CISH* knockdown can only promote the expression of *CIDEC* and *FASN* (Figure 6F). In ICP cell, *SOCS2* overexpression significantly inhibited the expression of fat deposition-related DEGs, whereas *CISH* overexpression can only inhibit the expression of *DGAT2* and *ApoB* (Figure 6G). The knockdown of *SOCS2* promoted the expression of most of the genes involved in fat deposition, whereas *CISH* knockdown only promoted *ApoA4* and *ApoB* expression (Figure 6H). To investigated whether *SOCS2* and *CISH* can affect lipid droplet accumulation during ICP differentiation, we transfected the overexpression vectors or siRNA of these two genes to the ICP and then induced the cells to differentiation for 48 h. As judged by Oil Red O staining and extraction assays, *SOCS2* overexpression significantly reduced lipid droplet accumulation (Figure 6I), whereas *SOCS2* inhibition significantly increased lipid droplet accumulation (Figure 6J). However, the overexpression and inhibition of *CISH* have no significant impacts on lipid droplet accumulation (Figures 6I,J). Taken together, these data indicate that *SOCS2* inhibits lipid metabolism and decreases lipid droplet accumulation.

### The Co-overexpression of *SOCS2* and *CISH* Rescues GHR Mutation-Induced Lipid Metabolism Disorder and Lipid Droplet Accumulation

**FIGURE 7:**
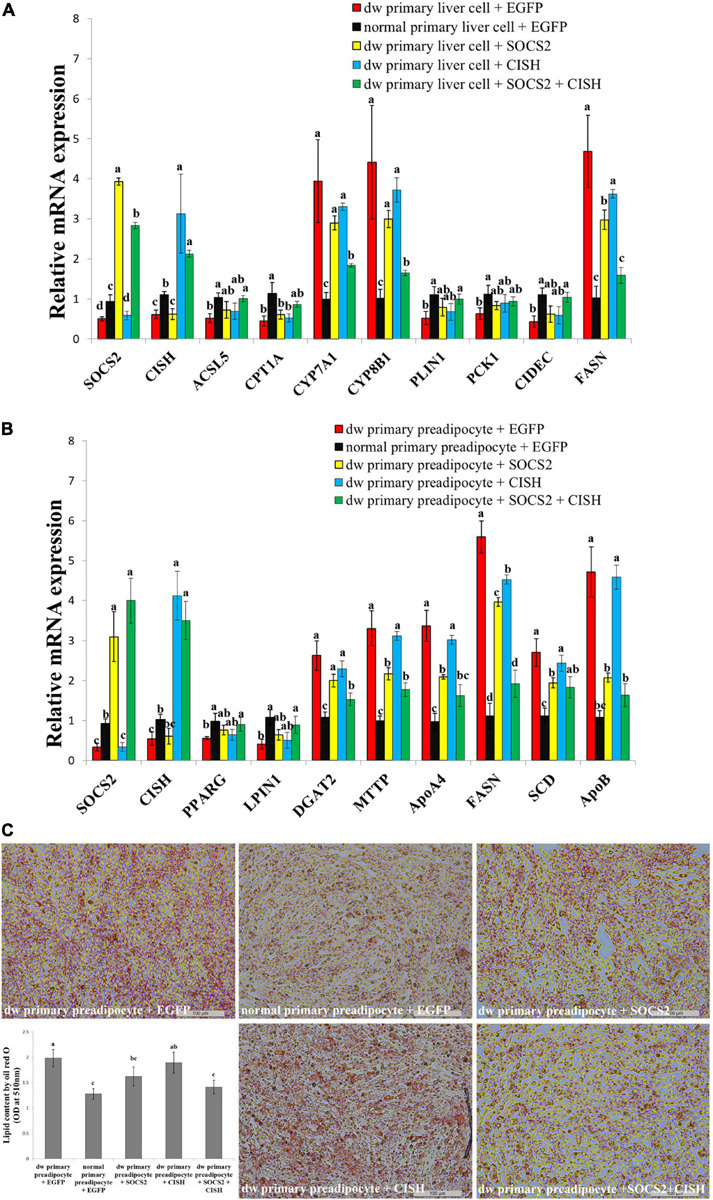
The co-overexpression of *SOCS2* and *CISH* rescues *GHR* mutation-induced lipid metabolism disorder and lipid droplet accumulation. **(A)** The relative mRNA expression of lipid metabolism genes after transfection of *SOCS2* and *CISH* overexpression vector in primary liver cells isolated from the sex-linked dwarf chicken and normal chicken. **(B)** The relative mRNA expression of fat deposition genes after transfection of *SOCS2* and *CISH* overexpression vector in primary preadipocytes isolated from the sex-linked dwarf chicken and normal chicken. **(C)** Representative images of Oil Red O staining (red) after the transfection of *SOCS2* and *CISH* in primary preadipocyte isolated from the sex-linked dwarf chicken and normal chicken; scale bar: 100 μm. The results are shown as the mean ± SEM of three independent experiments. Different letters a–d above the bars indicate significant differences (*P* < 0.05) by Duncan’s multiple range test.

*SOCS2* and *CISH* were both downregulated in the liver and fat of the SLD chicken compared with normal chicken (Figures 6A–D). In order to explore the important roles of *SOCS2* and *CISH* on *GHR* mutation-induced fat deposition, we used primary liver cell and preadipocyte isolated from the SLD chicken and normal chicken, respectively, to test the function of *SOCS2* and *CISH* on lipid metabolism and lipid droplet accumulation. In accordance with the RNA-seq data, the expression of *SOCS2*, *CISH*, and DEGs involved in lipid metabolism and fat deposition was dysregulated in primary cells from the SLD chicken (Figures 7A,B). Overexpression of *SOCS2* or *CISH* alone in the cells from the SLD chicken can partly rescue the dysregulated expression of genes involved in lipid metabolism and fat deposition, whereas co-overexpression of *SOCS2* and *CISH* can rescue most of the dysregulated expression of these genes (Figures 7A,B). Importantly, primary preadipocyte isolated from the SLD chicken can accumulate more lipid droplet than that from the normal chicken (Figure 7C). Overexpression of *SOCS2* can significantly decrease lipid droplet accumulation in preadipocyte from the SLD chicken, whereas no significant alteration was observed after transfection of *CISH* relative to control. However, co-transfection of *SOCS2* and *CISH* decreased more lipid droplet accumulation than individual transfection of *SOCS2*, suggesting a complementary role between *SOCS2* and *CISH* (Figure 7C). Taken together, these results indicate that co-overexpression of *SOCS2* and *CISH* can make the lipid droplet accumulation of the SLD chicken return to the level of normal chicken, suggesting that *SOCS2* and *CISH* are important in *GHR* mutation-induced fat deposition in the SLD chicken.

## Discussion

The broilers used in our study were SLD chicken, which was caused by missense mutation in *GHR* exon 5. The recessive mutation of *GHR* results in the disruption of the affinity between GH and GHR protein and further inactivation of the GH–GHR–IGFs growth axis, leading to short stature, weight loss, and obesity (Carter-Su et al., 1996). The SLD chicken is widely used in the broiler industry due to its relatively higher feed conversion, higher egg laying performance, and better meat quality (Guillaume, 1976). However, excessive fat deposition is frequently found in the SLD chickens, causing a series of negative effect, such as feed conversion rate reduction and depressed reproduction performance. The precise molecular mechanism of *GHR* mutation-induced excessive fat deposition in chicken remains unknown, though it has been shown that *GHR* mutation impedes lipolysis and fat deposition through the GH–GHR–IGFs axis (Guevara-Aguirre and Rosenbloom, 2015). Therefore, it is worth to investigate the regulatory genes and pathways of excessive fat deposition of the SLD chicken. In this study, the phenotype of the SLD chicken under an HFD and normal diet was determined. It was found that the chickens have no significant difference in body weight under the HFD and the normal diet, but the body weight of the SLD chicken was lower than that of normal chicken. The abdominal fat weight and abdominal fat rate of the SLD chicken were significantly higher than those of normal chicken in both diets, suggesting that the ability of fat deposition of the SLD chicken was greater than that of normal chicken, similar to *GHR* mutation mice (Erman et al., 2011; Zhang et al., 2018). On the other hand, the adipocyte cell size of the SLD chicken was larger than that of normal chicken. Notably, HFD can increase the abdominal fat weight, abdominal fat rate, and adipocyte size of both the SLD chicken and normal chicken. The accumulation of fat comes from the increase of adipocyte number and adipocyte size. Our results have shown that the adipocyte size of the SLD chicken was larger than that of normal chicken, but the number of adipocytes was hard to count. Considering that the genes related to adipogenesis were highly expressed in the SLD chicken, we believe that both the size and number of adipocytes in the SLD chicken were larger than those in normal chicken. Besides, HFD can increase the abdominal fat weight and adipocyte size of both the SLD chicken and normal chicken. However, as shown in Figure 1B, the increase scale in normal chicken (about four times) was larger than that in the SLD chicken (about 1.7 times). A similar phenomenon was also observed in *GHR*^–/–^ mice. Under the HFD, the fat weight of wild-type (WT) mice was increased by 3.1 times, whereas the fat weight of *GHR*^–/–^ mice was only raised by 1.9 times (Berryman et al., 2006). Therefore, *GHR* mutation or knockout not only increased fat deposition but also limited excessive fat deposition caused by HFD.

Many genes have been found to be involved in the regulation of lipid metabolism and fat deposition, such as *ACSL5* (Oikawa et al., 1998), *CPT1A* (Schlaepfer and Joshi, 2020), *CYP7A1* (Chen et al., 2012; Hori et al., 2020), *CYP8B1* (Kwong et al., 2015; Hori et al., 2020), *PLIN1* (Sun et al., 2020), *PCK1* (Xu et al., 2020), *CIDEC* (Zhou et al., 2012), and *FASN* (Wallace and Metallo, 2020), which play key roles in fatty acid metabolism in the liver. On the other hand, many genes are essential for lipid biosynthesis in the abdominal fat, such as *PPARG* (Lehrke and Lazar, 2005; Semple, 2006), *LPIN1* (Csaki et al., 2014; Chen et al., 2015), *DGAT2* (Bhatt-Wessel et al., 2018), *MTTP* (Jamil et al., 1995; Iqbal et al., 2020), *ApoA4* (Wang et al., 2019), *FASN* (Wallace and Metallo, 2020), *SCD* (ALJohani et al., 2017; Liu et al., 2020), and *ApoB* (Sirwi and Hussain, 2018). However, it is still unclear which lipid metabolism-related gene would be directly affected by the *GHR* gene mutation and then cause abdominal fat deposition in the SLD chickens. In this study, we found that *SOCS2* and *CISH* are two important genes that mediated *GHR* signaling and lipid metabolism-related genes and pathways. The expression of *SOCS2* and *CISH* was downregulated in *GHR*-downstream network analysis, and the function of these two genes was related to *GHR* mutation-induced fat deposition. *SOCS2* and *CISH* are members of SOCS protein and CISHs family, respectively. Most SOCS proteins are regulated by cytokine and form negative regulatory pathways with cytokine signals (Davies et al., 2007). Among *GHR*-downstream networks, *SOCS* negatively regulated *JAK2* and *STAT3*, whereas *CIS* negatively regulated *STAT1* (Krebs and Hilton, 2001). SOCS proteins are involved in a wide range of physiological processes (Yoshimura et al., 2007). It has been shown that *SOCS1* and *SOCS3* are involved in the induction of insulin resistance, and that the ablation of *SOCS3* expression in adipose tissue of female mice improves insulin sensitivity to obesity (Palanivel et al., 2012). In 3T3L1 adipocytes, the *SOCS3* expression is increased by insulin, which can bind to the insulin receptor and inhibit *STAT5b* expression (Emanuelli et al., 2001). Besides, the expression of *SOCS1* or *SOCS3* can depress the activation of glucose uptake in 3T3L1 cells, and both negatively regulate insulin signal pathway (Ueki et al., 2004). However, *SOCS2* plays a different role from other family members. SOCS2 protein can form E3 ubiquitin ligase with Elongin B/C to ubiquitinate SOCS3 (Tannahill et al., 2005). *CISH* is the first uncover gene in the *SOCS* family. The variation in *CISH* results in the change of human sensitivity to infectious disease (Khor et al., 2010). Conditional knockout of *CISH* in beta cells confirms that *CISH* and *SOCS2* negatively regulate the proliferation and function of beta cells (Jiao et al., 2013). *CISH* mRNA expression was increased in differentiated 3T3-L1 cells stimulated by GH, and mice fed an HFD had higher intra-abdominal adipose tissue and a higher expression of *CISH* than control mice (Hayashi et al., 2017). In addition, SOCS/CISH proteins may serve as part of the feedback loop to stimulate lipolysis, *via* inhibiting the JAK/STAT signaling pathway (Moller and Jorgensen, 2009). In this study, the roles of *SOCS2* and *CISH* on lipid metabolism were studied. We found that the mRNA expression of *SOCS2* and *CISH* in primary liver cell and preadipocyte of the SLD chicken was suppressed, and that the overexpression of *SOCS2* and *CISH* can depress the expression of genes relative to fatty acid metabolism, adipocyte differentiation, and lipid droplet accumulation, suggesting their effect on adipogenesis.

The effects of interaction between the *SOCS2* and GH–GHR axes on growth and fat deposition have been reported in several studies. *SOCS2*-deficient mice displayed an increase of organ and bone after birth, though there was no significant change at birth. The body weight of *SOCS2* specific-knockout mice was increased by more than 30%, and exogenous GH supplementation can reduce the overgrowth of *GH* and *SOCS* DKO mice (Greenhalgh et al., 2005). SOCS2 can bind phosphorylated tyrosine of GHR and negatively regulate GH signal (Greenhalgh et al., 2005). The overgrowth of mice with abnormal *SOCS2* expression seems to be caused by the failure of depression mediated by *GHR*, leading to the continued expression of *STAT5* (Turnley, 2005). In the liver of *SOCS2* knockout mice, the expression of *GHR* was increased, and in turn, *SOCS2* regulates the cellular expression of *GHR* by the way of ubiquitylation (Vesterlund et al., 2011). The *SOCS2* knockout mice showed an increase of 77.6% in hepatic triglycerides, whereas the hepatic steatosis caused by HFD was depressed. Under the HFD, *SOCS2* knockout mice showed a significant decrease in triglycerides of the liver (Zadjali et al., 2012). In the same way, the overexpression of *SOCS2* in porcine primary adipocyte can significantly depress the expression of *PPAR*γ, *FAS*, and *ATGL* and suppress *STAT3* and *STAT5*, suggesting that *SOCS2* may be an important regulator of GH signal in porcine adipocyte (Yang et al., 2012). In this study, the overexpression of *SOCS2* can depress the genes related to lipogeneses and fat deposition. In addition, we also found that co-transfection of *SOCS2* and *CISH* can rescue hyperactive lipid metabolism and fat deposition. It was shown that *SOCS2* and *CISH* interact with the leptin receptor. SOCS2 binds to the Y1077 motif of leptin and has a higher affinity to mediate the combination of CISH and STAT5a to this site (Lavens et al., 2006). In the liver of mice, GH activated the expression of *SOCS2* and *CISH*. Co-transfection assay suggested that *CISH* can depress the activation of *STAT5* mediated by GH (Karlsson et al., 1999). CISH regulates GHR internalization through proteasome mechanism and ultimately downregulates GH signal (Landsman and Waxman, 2005). In the SLD chicken, the detailed regulatory mechanism of interaction between *SOCS2* and *CISH* on lipid metabolism and fat deposition still needs to be further explored.

An HFD can effectively increase abdominal fat deposition and body weight. For the two chicken breeds in our study, abdominal fat weight was significantly increased under HFD, whereas body weight was not significantly affected, suggesting that the excessive energy of the HFD was mainly used for fat storage. The HFD can increase the level of free fatty acid, change energy dynamic balance, and affect brain cognition in a short period (Holloway et al., 2011). Long-term high-fat intake promotes fat accumulation more than consumption, which leads to obesity, diabetes, and fatty liver disease (Hariri and Thibault, 2010; Saponaro et al., 2015). The different levels of energy intake can lead to the distinct expression of *GHR* and downstream signaling in subcutaneous adipose tissue. After long-term energy restriction, the expression of *GHR* significantly increased, and *STAT3* expression decreased, whereas a long-term overfeeding led to an opposite expression trend (Glad et al., 2019). In the SLD chicken, this kind of GH-mediated signaling change resulting from alternation of diet energy content may be disrupted. On the other way, GHR-KO mice exhibit an excessive fat deposition, but they have the ability to resist metabolic deterioration inducted by HFD and reduce cancer risk (Dehkhoda et al., 2018). Presently, the mechanism of reduced side effects of feeding an HFD in the *GHR* mutation model remains to be studied.

## Conclusion

To summarize, our results show that the SLD chicken has higher abdominal fat rate and larger adipocyte size than normal chicken, and that an HFD can increase the abdominal fat rate and adipocyte size in chicken. Integrative analysis of the gene expression profiles of livers and abdominal fats between the SLD chicken and normal chicken revealed that many DEGs are associated with cell growth, lipid metabolism, lipid transport, and adipocyte differentiation pathways. Moreover, our results suggest that *SOCS2* and *CISH* are the core regulators for *GHR* mutation-induced fat deposition in the SLD chicken.

## Data Availability Statement

The datasets presented in this study can be found in online repositories. The names of the repository/repositories and accession number(s) can be found in the article/Supplementary Material.

## Ethics Statement

The animal study was approved by the Institutional Animal Care and Use Committee of the South China Agricultural University.

## Author Contributions

WL, QN, and XZ conceived and designed the study. WL and GC drafted the manuscript. GC, JC, XR, LL, SLu, TC, and LT performed the experiments. WL, GC, JC, JW, XR, ML, SLi, and QL carried out the data analysis. All authors have read and approved the final manuscript.

## Conflict of Interest

The authors declare that the research was conducted in the absence of any commercial or financial relationships that could be construed as a potential conflict of interest.

## Footnotes

1 http://www.ncbi.nlm.nih.gov/geo

2 http://bioinformatics.psb.ugent.be/webtools/Venn/

3 http://metascape.org/gp/index.html#/main/step1

4 http://www.kegg.jp/kegg/kegg1.html

## Supplementary Material

Supplementary File 1 The specific primers used for plasmid construction and qRT-PCR.

Supplementary File 2 The genes list of scatter plot of differentially expressed genes (DEGs) between ND and NN chicken in the liver.

Supplementary File 3 The genes list of scatter plot of DEGs between HD and HN chicken in the liver.

Supplementary File 4 The distribution of DEGs between ND-vs-NN and HD-vs-HN in the liver.

Supplementary File 5 The genes list of scatter plot of DEGs between ND and NN chicken in the fat.

Supplementary File 6 The genes list of scatter plot of DEGs between HD and HN chicken in the fat.

Supplementary File 7 The distribution of DEGs between ND-vs-NN and HD-vs-HN in the fat.

Supplementary File 8 The distribution of DEGs between HD-vs-ND and HN-vs-NN in the liver.

Supplementary File 9 The distribution of DEGs between HD-vs-ND and HN-vs-NN in the fat.

Supplementary File 10 The distribution of DEGs among the four treatment groups.

## Abbreviations

AACS: acetoacetyl-CoA synthetase
ACC: anterior capsular cataract
ACSL5: acyl-CoA synthetase long-chain family member 5
AKT: serine/threonine kinase
AOX: acyl-coenzyme A oxidase 1, palmitoyl
ApoA4: apolipoprotein A4
ApoB: apolipoprotein B
ATG1: serine/threonine–protein kinase
Bcl-XL: BCL2 like 1
CIS: cytokine inducible SH2 containing protein
c-fos: Fos proto-oncogene, AP-1 transcription factor subunit
CIDEC: cell death inducing DFFA like effector c
c-Myc, v-myc: avian myelocytomatosis viral oncogene homolog
CPT1A: carnitine palmitoyltransferase 1A
CREB: cAMP responsive element binding protein
Cycd: Cyclin D
CYP7A1: cytochrome P450 family 7 subfamily A member 1
CYP8B1: cytochrome P450 family 8 subfamily B member 1
DGAT2: diacylglycerol O-acyltransferase 2
4E-BP: eukaryotic translation initiation factor 4E binding protein
eIf4E: eukaryotic translation initiation factor 4E
ERK1/2: mitogen-activated protein kinase 3/1
ETNPPL: ethanolamine-phosphate phospho-lyase
FAS: Fas cell surface death receptor
FASN: fatty acid synthase
FBP: fructose-1,6-bisphosphatase
FOXO: forkhead box O
G6PC: glucose-6-phosphatase catalytic subunit
GFAP: glial fibrillary acidic protein
GK: glycerol kinase
GRB2: growth factor receptor bound protein 2
Grb10: growth factor receptor bound protein 10
GSK3B: glycogen synthase kinase 3 beta
IGF1: insulin-like growth factor 1
IGF1R: insulin-like growth factor 1 receptor
IGF2: insulin-like growth factor 2
IGF2BP1: insulin-like growth factor 2 mRNA binding protein 1
IKK: I-kappaB kinase
IL21R: interleukin 21 receptor
IRS: isoleucyl-tRNA synthetase 1
JAK2: Janus kinase 2
JNK: mitogen-activated protein kinase 8
JunD: JunD proto-oncogene
AP-1: transcription factor subunit
LBFABP: liver basic fatty acid binding protein
LPIN1: lipin 1
MCL1: BCL2 family apoptosis regulator
MEK1/2: mitogen-activated protein kinase kinase 1/2
MEKK1: mitogen-activated protein kinase kinase kinase 1
MKK4: mitogen-activated protein kinase kinase 4
Msx1: msh homeobox 1
mTOR: mechanistic target of rapamycin
MTTP: microsomal triglyceride transfer protein
NSDHL: NAD(P)-dependent steroid dehydrogenase-like
p21: cyclin dependent kinase inhibitor 1A
p70S6K: ribosomal protein S6 kinase B1
PCK1: phosphoenolpyruvate carboxykinase 1
PDK1: pyruvate dehydrogenase kinase 1
PEPCK: phosphoenolpyruvate carboxykinase 1
PI3K: phosphatidylinositol 3-kinase
PIM1: Pim-1 proto-oncogene, serine/threonine kinase
PKC: protein kinase C
PLIN1: perilipin 1
Plk: polo like kinase
PPARG: peroxisome proliferator-activated receptor gamma
PTEN: phosphatase and tensin homolog
PYK: pyruvate kinase
RAS: resistance to audiogenic seizures
RAF: Raf-1 proto-oncogene, serine/threonine kinase
SCD: stearoyl-CoA desaturase
SHC: SHC adaptor protein
SLN: sarcolipin
SOCS2: suppressor of cytokine signaling 2
SOS: Ras/Rac guanine nucleotide exchange factor 1
SREBP-1c: sterol regulatory element binding protein-1c
STAT1: signal transducer and activator of transcription 1
STAT3: signal transducer and activator of transcription 3
STAT5: signal transducer and activator of transcription 5.
