# Supplementary material for: Integrative Analyses of mRNA Expression Profile Reveal SOCS2 and CISH Play Important Roles in GHR Mutation-Induced Excessive Abdominal Fat Deposition in the Sex-Linked Dwarf Chicken
Source: Front Genet. 2021 Jan 14;11:610605. doi: 10.3389/fgene.2020.610605 (PMC7841439; doi:10.3389/fgene.2020.610605)
Supplement: Supplementary File 10 — The distribution of DEGs among the four treatment groups. [file Data_Sheet_1.DOCX]

**For cloning the following primers were used for PCR:**

*SOCS2* CDS (reference sequence:) cloning for pcDNA3.1 overexpression vector:

s: 5’- cgGGATCCATGCGTCGTAAGGCAAATTATTTTC

as: 5’- cgGAATTCAACCTTTCCCCCAGAACTGC

CISH CDS (reference sequence:) cloning for pcDNA3.1 overexpression vector:

s: 5’- cgGGATCCATGATCCTCTGCGTCCCGGGACC

as: 5’- cgGAATTCAAGGACACGCCAAGTTCTCT

**For quantitative real time RT-PCR, the following primers were used:**

NSDHL-qPCR (reference sequence: XM_420279.5):

s: 5’- GGTGGCTCCGGATTCTTAGG

as: 5’- AGCTGGGAGTAAGGCCTCTT

AACS-qPCR (reference sequence: NM_001006184.1):

s: 5’- ATGGTTCATTCAGCAGGGGG

as: 5’- CCAGCCAGTCGTTGTGTAGT

ETNPPL-qPCR (reference sequence: XM_015276580.2):

s: 5’- GGAGGAGCGCTACAGCAAGG

as: 5’- AACTTTGCAGGAAGGCCCGAT

DGAT2-qPCR (reference sequence: XM_419374.6):

s: 5’- CACGGGAGTCAGCCAGAAAT

as: 5’- TCACGGGACATATACCCCCA

IL21R-qPCR (reference sequence: NM_001030640.1):

s: 5’- TGCGGTCCCAGTAACCATTT

as: 5’- CTGACTGGATGTCCTTGCCC

SLN-qPCR (reference sequence: NM_001302187.1):

s: 5’- GTATCTGCAGTGCCCCAGAG

as: 5’- GGTCACAGGCTCTTATGAAGGT

LBFABP-qPCR (reference sequence: NM_204634.1):

s: 5’- TCTGAAAGCTCTTGCACTGCC

as: 5’- ACAGTCTGCCTGGGTGTTTT

Msx1-qPCR (reference sequence: NM_205488.2):

s: 5’- AAGCAGTACCTGTCCATCGC

as: 5’- AGATCTTCACCTGCGTCTCG

SOCS2-qPCR (reference sequence: NM_204540.1):

s: 5’- GTACCAGGACGGCAAGTTCA

as: 5’- GCTGTTGAACTGCTTGAGCC

CISH-qPCR (reference sequence: NM_204626.1):

s: 5’- GCTGGCAGAGGAGAAGATCC

as: 5’- AGTACCAGCCGGACTCCC

LPIN1-qPCR (reference sequence: XM_015276089.2):

s: 5’- CCTGTGCAAAGAATGGGGGA

as: 5’- CTTGCCCTGCTAACTGTCCA

MTTP-qPCR (reference sequence: NM_001109784.2):

s: 5’- TTCTGAAGGACATGCGTGCT

as: 5’- GTCTAGGCCGTACGTGGATG

APOA4-qPCR (reference sequence: NM_204938.2):

s: 5’- GCACTCAGGATGTCGCCTAA

as: 5’- CGGTGAAGTACCTCCAGAGC

SCD-qPCR (reference sequence: NM_204890.1):

s: 5’- ACCTTAGGGCTCAATGCCAC

as: 5’- TCCCGTGGGTTGATGTTCTG

APOB-qPCR (reference sequence: NM_001044633.1):

s: 5’- GGTTACTCCCACGATGGCAA

as: 5’- AATGCCCTTCCTTCAGGAGC

ACSL5-qPCR (reference sequence: NM_001031237.1):

s: 5’- CCCTAAAGGTGCCATGCTGA

as: 5’- CTCCGCAGCTGTACATCACA

CPT1A-qPCR (reference sequence: NM_001012898.1):

s: 5’- ACAGCGAATGAAAGCAGGGT

as: 5’- GCCATGGCTAAGGTTTTCGT

CYP7A1-qPCR (reference sequence: NM_001001753.1):

s: 5’- GTAACGCCCTAGATGCCCTC

as: 5’- GCTCTCTCTGTTTCCCGCTT

CYP8B1-qPCR (reference sequence: NM_001005571.1):

s: 5’- CATCATTCCCTGGCTGGGTT

as: 5’- TAGCCAAAAACCCGGAGGAC

FASN-qPCR (reference sequence: NM_205155.3):

s: 5’- GCTAAGATGGCATTGCACGG

as: 5’- TGCCAGAGCCTCCACTATCT

IGF2BP1-qPCR (reference sequence: NM_205071.1):

s: 5’- AAGGCACAAGGCAGGATT

as: 5’- GCAGCTCATTGACGGTTTT

PPARγ-qPCR (reference sequence: NM_001001460.1):

s: 5’- TACATAAAGTCCTTCCCGCTGACC

as: 5’- TCCAGTGCGTTGAACTTCACAGC

PCK1-qPCR (reference sequence: NM_205471.1):

s: 5’- CCAGATAATGGGGAGCCGTG

as: 5’- CAGGTCTGCGACCTCCAAAT

CIDEC-qPCR (reference sequence: NM_001277678.1):

s: 5’- GGTGTAGGCTCAGTTCCGT

as: 5’- TATTTGGAGACAGGCGCAGC

PLIN1-qPCR (reference sequence: NM_001127439.1):

s: 5’- ATGGAAGGGCCAAGGAGAAC

as: 5’- CCCGACATGCCAAAGTGTTG

GAPDH-qPCR (reference sequence: NM_204305.1):

s: 5’- AGAACATCATCCCAGCGT

as: 5’- AGCCTTCACTACCCTCTTG

β-actin-qPCR (reference sequence: NM_205518.1):

s: 5’- TTGTTGACAATGGCTCCGGT

as: 5’- AACCATCACACCCTGATGTCT
